# Nitric oxide generating/releasing materials

**DOI:** 10.4155/fso.15.54

**Published:** 2015-08-01

**Authors:** Hongying Liang, Parimala Nacharaju, Adam Friedman, Joel M Friedman

**Affiliations:** 1 Department of Physiology & Biophysics, Albert Einstein College of Medicine, 1300 Morris Park Ave, Bronx, NY 10461, USA; 2 Department of Dermatology, George Washington School of Medicine & Health Sciences, NW, Washington, DC 20037, USA

**Keywords:** liposomes, nanoparticles, nitrosothiols, NO, NONOates

## Abstract

Harnessing the impressive therapeutic potential of Nitric oxide (NO) remains an ongoing challenge. This paper describes several of the current strategies both with respect to the underlying chemistry and physics and to the applications where they have shown promise. Included in this overview are molecular systems such as NONOates that release NO through chemical reactions and delivery vehicles such as nanoparticles that can generate, store, transport and deliver NO and related bioactive forms of NO such as nitrosothiols. Although there has been much positive movement, it is clear that we are only at the early stages of knowing how to precisely produce, transport and deliver to targeted sites therapeutic levels of NO and related molecules.

**Figure 1.**
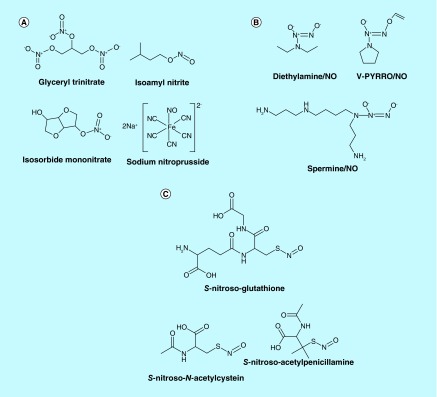
**Nitric oxde donor drugs.** **(A)** Some nitrates/nitrite/nitroso products. Glyceryl trinitrate, isoamyl nitrite. Isosorbide mononitrate, sodium nitroprusside. **(B)** Some diazeniumdiolates. Diethylamine/NO V-PYRRO/NO. Spermine/NO. **(C)** Some *S*-nitrosothiols. *S*-nitrosoglutathione; *S*-nitroso-*N*-acetylcysteine; *S*-nitroso-acetylpenicillamine. NO: Nitric oxide.

**Figure 2.**
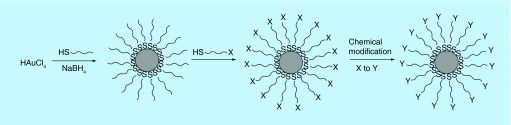
**Synthesis of gold nanoparticles.** HAuCl4 is reduced by sodium borohydride in the presence of a stabilization agent, a thiol (-SH) ligand. The thiol ligand is exchanged by another thiol reagent carrying a desired functional group (x) that can be derivatized into a final product (y).

**Figure 3.**
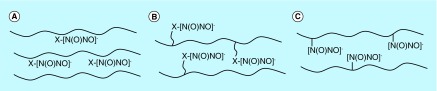
**Three different approaches used to incorporate nitric oxide donors within polymer materials.** NONOate groups are noncovalently dispersed within the **(A)** polymer matrix, **(B)** covalently bound to pendent polymer side chains, **(C)** covalently bound directly to the polymer backbone.

**Figure 4.**
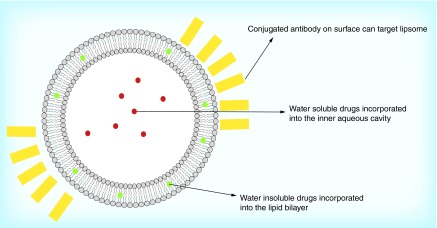
**Liposome structure.**

Nitric oxide (NO) is a free radical that is naturally produced in mammals. It has been well known to play a key role in a wide variety of physiological and pathophysiological important processes, such as neurotransmission, neuronal communication, platelet adhesion, thrombosis, vasodilation, inflammation and wound healing [1]. Over the past two decades, countless research has been conducted to develop most effective NO-generating and NO-releasing materials for clinical therapies. A great number of synthetic compounds (e.g., N-diazeniumdiolates, nitrosothiols, nitrosohydroxylamines and nitrosyl metal complexes) have been developed to chemically stabilize and release NO in a controlled manner, and have been exploited in many biomedical applications. The translation of the therapeutic potential of NO to bedside has been slowed down by its short biological lifetime, instability during storage and potential toxicity. A promising strategy for addressing these limitations and thus increasing the targeted NO release is developing NO-delivery devices/vehicles based on nanotechnology.

## NO donors

### Nitrate/nitrite/nitroso compounds

Organic nitrates and nitrites are the NO donor drugs currently in use to treat coronary artery disease. Nitroglycerin (glyceryl trinitrate), isosorbide dinitrate, isosorbide mononitrate and isoamyl nitrite are the examples of this class (Figure 1A). These products release NO on exposure to certain endogenous enzymes [2]. Extended use of nitrate vasodilators induce nitrate tolerance leading to tachyphylaxis as well as aggressive side effects including increased oxidative stress, endothelial dysfunction and cardiac autonomic dysfunction [1,3]. The tolerance is dependent on the type of nitrate and dosing schedule [4]. Slow release forms of isosorbide mononitrate and dinitrate have been formulated to reduce nitrate tolerance [5]. Supplementing the nitrate treatment with antioxidants seems to reduce the oxidative stress-induced damage [3,6–8].

Sodium nitroprusside (SNP) is another NO-based drug that is in use for vasodilation in emergency setting to treat acute hypertension [9]. SNP is a rapid vasodilator with a very short half life, needing rapid titration. The major complications of SNP therapy are hypotension, and toxicity from accumulation of cyanide, usually in patients with renal insufficiency, when treated for more than 24 h [10,11].

### N-diazeniumdiolates

Diazeniumdiolates (NONOates) are another class of NO donors that are getting great attention recently for the ease of preparation and generating predictable amounts of NO from these products [2,12]. NONOates carry an [N(O-)N=O] group on a nucleophile adduct, usually an amine (Figure 1B) [13,14]. NONOates decompose spontaneously in solution at physiological pH and temperature to generate NO. The rate of decomposition is solely dependent on the structure of the nucleophile, pH and temperature of the medium and not influenced by reducing agents or biological tissue [12,14]. The biological activity of these products correlate with the amount of NO released [14]. By structural manipulations, NONOates can be designed for targeted delivery of NO [2,12,15–16] and conjugate other therapeutic molecules to enhance their therapeutic potential [2,17–18]. Since the NO release from NONOates is not influenced by biological factors, these products do not develop tolerance [19].

The biological activity of NONOates has been tested in various experimental models such as vasospasm, pulmonary hypertension, platelet adhesion/aggregation, with promising results [2,12,20]. Saavedra *et al*. [15] designed a prodrug V-PYRRO/NO for targeted delivery of NO to liver. This drug releases NO only after being processed by CYP450. The same group also developed another NONOate prodrug that would release NO only following esterase activity within cells, causing apoptosis of the human leukemia cell lines studied [21]. Tang *et al*., [16] coupled PYRRO/NO to a chain of amino acids recognized to be a substrate for prostate-specific antigen, which is upregulated within prostate cancer metastases. Conjugation of antitumor agent, 5-fluorouracil, to an NONOate enhanced the cytotoxicity effect of the conjugate on cancer cells [17].

NONOates have also been tested in human subjects. Inhaled DETA/NO reduced pulmonary vascular resistance without affecting the systemic blood pressure or cardiac output in patients with acute respiratory distress syndrome [22,23].

At present, NONOates are not yet approved for clinical use. However, the possibility of modulation of structure to control NO release as well as for targeted delivery, enabling tagging other therapeutic molecules and lack of tolerance make NONOates potential candidates for therapeutic use. The toxicity of NONOates has not yet been fully established. Accumulation of amines and their metabolites released from NONOates can induce cytotoxicity [12,20]. More detailed studies are needed to establish long-term safety of these products.

### *S*-nitrosothiols

*S*-nitrosothiols (RSNOs) are adducts of R-SH and NO and considered as excellent source of NO (Figure 1C). The rate of NO release from RSNOs is influenced by various factors, metal ions, reducing agents and enzymes, light, heat and pH [2,24]. Endogenous RSNOs such as *S*-nitrosoglutathione (GSNO) are distributed in red blood cells, plasma and tissue at different concentrations [2,25]. RSNO-based therapeutics exhibit physiologic effects very similar to other NO-related therapeutics [24,26–28]. RSNOs have also been tested in humans as therapeutics for different clinical conditions with promising results [2].

RSNO-based therapeutics can be considered more efficient than NO-based therapeutics due to their capacity for long lasting release of NO as well as a more facile transnitrosating capability. SNO-based NO donors have been shown to induce vasodilation longer than other types of NO donors tested [29,30]. Lipophilic *S*-nitrosothiols induced vasodilation even longer than conventional S-nitrosothiols [2].

Since the NO release from RSNOs depend on various physiological conditions and does not depend on a specific mechanism like nitrate, these products do not induce tolerance on long-term use [2,31–33]. The long lasting biological effects of RSNOs demonstrated in preclinical trials make them potential therapeutics for NO-/SNO-based drugs. The recent findings on the role of *S*-nitrosothiols in various biological processes promote further the exploration/urge of developing RSNO-based drugs.

## Vehicles for NO donors

### Gold nanoparticles

Gold nanoparticles (GNP) are nontoxic, biocompatible and stable. The particles can be synthesized with desired size (2–250 nm) and surface functional groups for the conjugation of therapeutic products. These features made the particles versatile carriers of molecules for biological applications. GNP have been developed as vehicles for drugs and gene delivery as well as for imaging [34–36].

The synthesis of GNP involves the reduction of tetrachloroaurate (AuCl4-) with sodium citrate or sodium borohydride and in the presence of a stabilization agent (Figure 2). The chemistry of the stabilizing agent determines the surface properties and functionality of the particles. Citrate-capped particles carry negative charge on the surface and are taken up by cells easily [35]. GNP have great affinity for thiol ligands. The use of an alkanethiol as a stabilizing agent yields nanoparticles stabilized by a surface monolayer of alkanethiols [34]. These monolayer-protected clusters of GNP can be used as basic blocks for the preparation of vehicles to carry desired molecules for bioapplications. Using suitable bifunctional alkanethiols the functionality of the monolayer can be changed through a substitution reaction to conjugate molecules of interest on the surface of the GNP [34].

Surface coating of GNP with amines is the most widely used technique for the generation of delivery vehicles [34–36]. Amine groups can be readily derivatized by different chemistry for the conjugation of therapeutic molecules. Amine-coated GNP have been directly used for gene delivery without further modification. Amines are positively charged at physiological pH and therefore can bind negatively charged nucleic acids. Amine-coated GNP were able to bind DNA plasmids and delivered to 293T cells much more efficiently than the commonly used cationic polymer transfection agent polyethylenimine [35].

Rothrock *et al*. [37] developed *N*-diazeniumdiolate-based NO-releasing GNP using amine-carrying GNP. The release rate and total amount of NO released from *N*-diazeniumdiolate were controllable by varying the number and/or chemical structure of the amine agent used in the synthesis of the particles. RSNO-based NO-releasing GNP have been prepared by simply mixing borohydride-reduced GNP with RSNOs [38]. SNAP-GNP were taken up by human hepatic stellate cells without inducing any toxicity. These GNP reduced the cell proliferation and tube formation, indicating potential therapeutic use of the particles for chronic liver disease. Sudhesh *et al*. [39] prepared nitrobenzimidazole-capped GNP as a potential cancer therapeutic. The cytotoxic effect of these GNP on cervical cancer cell lines was much higher in the presence of light than in the absence indicating the role of released NO in the effect.

The biocompatibility of GNP is highly dependent on the stabilizing agents coated on the surface of GNP. Citrate-treated GNP have been shown to release NO from RSNOs *in vitro* as well as from serum samples [40]. The NO production from RSNOs was inhibited by coating GNP with glutathione. This property of GNP has been considered as a potential to induce oxidative stress and cytotoxicity in biological applications [40,41]. Amine-coated GNP can be cytotoxic due to the positive charge. Efforts are being made to generate more biocompatible GNP by derivatizing the surface amines to acetamides or hydroxyl groups [42].

### Silica nanoparticles

Sol–gel-derived materials serve as excellent carriers of molecules due to their porous structures. Sol–gel technology has been applied in biomedical applications, delivery of therapeutic drugs, development of biosensors and stationary phases for chromatography, biophysical studies to evaluate protein dynamics, protein–ligand interactions and protein–protein interactions [43,44]. The encapsulation of delicate biomolecules such as enzymes, antibodies and even whole cells could be safely trapped within sol–gel glasses. They retain their bioactivity and are protected by the silica cage [45,46].

The sol–gels are usually prepared from inorganic metal salts or metal organic compounds such as metal alkoxides. The most commonly made sol–gels are silica sol–gels. Silica nanoparticles (SiNP) developed from these sol–gels are considered as stable, nontoxic and biocompatible for delivering bioactive molecules [47,48]. Typically, SiNP are made from alkoxysilanes. Derivatives of silanes with different functional groups such as amines and thiols are commercially available. While the porous sol–gels made from alkoxysilanes alone are useful to simply load biomolecules into the pores, the derivatives of silanes offer multiple applications. The functional groups can be used to conjugate biomolecules to SiNP, which may not be efficiently retained by the porous structures of sol–gels. In addition, target molecules can be attached to the surface of particles for site-specific drug delivery. Polyethylene glycol chains may be surface coated to enhance the circulation life of SiNP.

In the recent years, SiNP have been developed for the delivery of NO from different NO donors such as nitrite, RSNOs and NONOates. An interesting variation on the silica particles is a hybrid nanoparticle platform [49] that has been shown to be highly effective for both topical [50–54] and systemic applications [47,55–56]. The use of the term hybrid refers to a combination of the standard silane-derived hydrogel with a strong amorphous hydrogen bonding network that confers a ‘glassy’ quality to the platform. The platform evolved out of the finding that nitrite could be converted to NO within sugar-derived glassy matrices through a solid state redox process that likely involves the formation of N_2_O_3_. The NO-containing glass rapidly dissolves upon contact with water releasing a burst of NO. The hybrid platform sought to use the hydrogel to provide a robust matrix and incorporate glassy (strong hydrogen bonding networks) elements that would allow for the solid state conversion of nitrite to NO and slow the release of NO. The resulting platform which uses chitosan to provide the internal ‘glassy’ element and thereby plug the pores of the hydrogel spontaneously forms nanoparticles that release NO in a slow sustained manner when the nanoparticles are exposed to moisture. Release rates of NO can easily be tuned through straightforward variation of the initial composition of the nanoparticles. More recently this platform has been used for systemic slow delivery of *S*-nitrosothiols [30].

NO-SiNP exhibited bactericidal activity in skin infections against drug-resistant bacteria (*Staphylococcus aureus*), promoted wound healing and reduced abscesses in murine models [54,57]. NO-SiNP also induced vasodilatory effects in various animal models [47,55–56]. However, NAC-SNO-SiNP induced longer vasodilatory effects than NO-SiNP in hamsters [30]. RSNOs are known to induce long-lasting vascular effects due to their capability of transnitrosation. NONOates-SiNP also exhibited antimicrobial activity as well as anticancer activity [48]. These results suggest that SiNP carrying NO donors may be useful for various NO-based therapeutic applications.

## Polymeric vehicles

Biocompatible polymer microparticles and nanoparticles have been used frequently as drug delivery vehicles due to their bioavailability, encapsulation capability, controlled release and low toxic properties [58]. Such polymers include PEG, polyurethane (PU), PMMA, poly(vinyl pyrrolidone) (PVP), poly(amidoamine), poly(ethylene oxide), poly(propylene oxide), poly(vinyl chloride), polylactic acid, polyglycolic acid and polylactic-co-glycolic acid. The NO donors can be incorporated into or chemically linked to biopolymers in order to mimic the endogenous NO production directly at the target site.

### Polymers

In the 1990s, Smith and coworkers [59] first successfully developed NO-releasing polyethyleneimine microspheres with NONOates as NO donors. They outlined three general polymer types (Figure 3) containing the NONOates. There is a wide range of control over the kinetics of NO release in these materials by varying the organic monomers used to prepare the polymers, which make them very promising candidates for therapeutic applications. Since then, research has focused on the preparation of new NO-releasing polymers and the application of these materials to biomedical devices. Here, the recent research on the polymer-based NO-releasing and -generating materials is discussed.

Polyurethanes are used as vascular substitutes. The enhanced blood compatibility of PU makes them particularly interesting in the scope of NO-releasing materials. Incorporation of a NONOate-tagged peptide into PU improved the thromboresistance of this polymer [60]. NONOate moieties can also be generated directly on the polymer chain of PU carrying secondary amines [61]. A new method of incorporation of methoxymethyl- or sugar-protected preformed NONOate moieties directly into chain extender diols, which are then incorporated into the PU backbone provided the ability to control the number of NONOate groups incorporated into the polymer backbone [62].

RSNOs have also been used as NO donors in numerous polymeric materials to form hydrogels and blended solid films [63,64]. GSNO-loaded solid films [58] of PVA/PVP blends stabilized GSNO and the films were capable of releasing both NO and GSNO to aqueous solution through diffusion. In another study [59], the authors covalently attached SNO groups to the polymer backbone, through the condensation reaction of diols (ethylene glycol and PEG) with mercaptosuccinic acid, followed by the S-nitrosation of the SH groups by a gaseous NO/O_2_ mixture. The polynitrosated polyesters provided sustained NO release for more than 20 h at physiological temperature.

DeRosa and coworkers [65] developed a new group of plastics that release NO. The NONOate derivatives of polyacrylonitrile have a duration of NO release >80 days. These NONOate-acrylonitrile-based polymers are stable at room temperature for many months and may thus represent a broad class of commercially viable materials to be developed as NO donors.

### Dendrimers

Dendrimers are monodisperse macromolecules with a tridimensional structure that is highly ramificated and regular around the nucleus. Stasko and Schoenfisch [66] prepared the NO release polypropylenimine dendrimers by generating NONOates on different amines. Secondary amine dendrimer conjugates exhibited high storage capacity for NO and released NO for longer periods than small molecule secondary amine NONOates. The authors further [67] synthesized two generation 4 poly(amidoamine) dendrimers with S-nitrosothiol exteriors and characterized their ability to inhibit thrombin-mediated platelet aggregation. Recently, Johnson *et al*. [68] developed a dendrimer with SNAP as NO donor. The NO release was initiated by glutathione. This system successfully reduced ischemia/reperfusion injury in an isolated, perfused rat heart with optimal therapeutic dose of NO donor under physiological glutathione concentrations.

### Micelles

Polymeric micelles are generally lower in size than nanoparticles and liposomes and larger than dendrimers, while sufficiently small to penetrate tissues. Jo and coworkers [69] designed block copolymer pro-amphiphiles and amphiphiles for providing long-term NO release. They suggested that the creation of a hydrophobic microenvironment within a micelle core could protect a NONOate from proton-catalyzed NO liberation. The hydrophobic core of the micelle shielded the NONOate from water, and thus protons, delaying NO release to a remarkable 7-day half life. Kanayama *et al*. [70] prepared PEGylated polymer micelles containing 4-nitro-3-trifluoromethylphenyl units as NO donors within the core. These micelles were capable of delivering exogenous NO into tumor cells in a photo controlled manner. Doung *et al*. [71] conjugated GSNO to copolymer backbone and used micellization approach to yield NO-polymeric nanoparticles. These polymeric nanoparticles substantially improved NO stability in aqueous media without affecting the efficacy of intracellular delivery.

The combined use of NO donors with polymeric matrices may not only favor a more controlled administration of the donor, but also regulate NO cellular effects. Sorragi *et al*. [72] found that PVA and PVP polymer solutions reduced the cytotoxicity and enhanced the antiproliferative effects of GSNO in smooth muscle cell cultures.

### Lipid-based nanocarriers

Liposomes are vesicles formed by an aqueous core surrounded by one or several lipid bilayers (Figure 4). Hydrophilic drugs can be incorporated into the inner aqueous cavity, while lipophilic drugs may be incorporated into the bilayer. Recently, liposomes have been used for carrying and delivering NO in various biomedical applications [73]. Huang *et al*. [74] developed a bioactive gas-delivery method, using echogenic liposomes as the NO carrier, to inhibit intimal hyperplasia. The cationic liposomes provide the highest NO delivery and incorporation into cells, with rapid NO release in the first 0.5 h, followed by slow NO release for the next several hours. RSNOs [75] and NONOates [76] have also been encapsulated into lipid vesicles. The basic intraliposomal environment dramatically delayed spontaneous NO release.

Recently, a prominent NO donor [Ru(terpy)(bdqi)NO](PF6)3 has been synthesized and bound to lipid carriers for topical administration [77]. The potential for delivering NO in therapeutic quantities is tenable since the nitrosyl ruthenium complex must first reach the ‘target tissue’ and then release the NO upon stimulus. Perera and coworkers [78] encapsulated nitric oxide synthase (NOS) enzymes within 1,2-distearoyl-glycero-3-phosphocholine with an encapsulation efficiency of about 25–40%. The NOS-loaded liposomes were stable for 15 days, during which the NOS-enzymatic activity was also retained. Ostrowski *et al*. [79] demonstrated that the photochemical NO precursors CrONO and mac-CrONO can be encapsulated into stable phosphatidylcholine liposomes and that photolysis of these in solution is an effective method for delivering NO. The liposomes provide a means to maintain a localized high concentration of NO-releasing complexes and are easily modified for *in vivo* targeting through self assembly.

## Safety & feasibility

The presented platforms all offer various benefits supported by several levels of evidence. From a translational standpoint, safety and practicality are the two greatest challenges. Even the clinically available NO-generating compounds, such as the organic nitrites/nitrates, have some limitations. Organic nitrates only release NO in tissues expressing the enzyme mitochondrial aldehyde dehydrogenase 2; however, this enzyme is irreversibly inactivated with use thus resulting in the emergence of tachyphylaxis (also known as nitrite/nitrate tolerance), thereby limiting the utility of this class of materials. Furthermore, continued use has been shown to upregulate PDE5, resulting in increased vasoconstriction; the complete opposite of the desired clinical outcome [80–82].

NONOate-associated toxicity is a topic of great concern, and a stimulus for modification. For example, V-PYRRO/NO has the potential to be converted to *N*-nitrosopyrrolidine, an extraordinarily potent experimental hepatocarcinogen [83]. In order to reduce the risk of the toxic metabolites, several groups have created NONOates bound to large immobile structures and nanoparticles. Sol–gel-based coatings which integrate N-(6-aminohexyl) aminopropyltrimethoxysilane can be loaded with NONOates. When the NO molecules are subsequently released, the metabolites remain bound to the sol–gel coating. However, even with the application of a surface coating, the *N*-nitroso groups are still cytotoxic to adjacent fibroblast cells at antimicrobial concentrations (40%) [84].

The utility of prepared *S*-nitrosothiols has limitations because thiols spontaneously form disulfide bonds in the presence of water and heat. Thus most *S*-nitrosothiols must be kept refrigerated as dry powder until they are mixed and administered, limiting translational applications [27].

Therefore, it is clear that successful utilization of an NO-donating agent will not only be based on the properties of the chemical compound itself, but that of the delivery vehicle and storage approach. As listed above, nanotechnology represents one such approach, but further innovation is needed to fully capitalize on the multifaceted benefits of NO therapy.

NO delivery via nanoparticles raises the added safety issue associated with the nanoparticles themselves. Most of the toxicity studies to date relating to NO delivery involve the silane- or silica-derived nanoparticles. No major toxicity has been observed to date. For the hybrid hydrogel nanoparticles both topical and systemic studies have been conducted. Topically applied hybrid hydrogel nanoparticles were shown not to cause any detectable inflammatory response in the surrounding skin in a rat model [85]. Systemically administered hybrid hydrogel NO-releasing nanoparticles were shown to be anti-inflammatory in a hamster model [47]. More recently, these nanoparticles were evaluated using a zebra fish embryo toxicity model [86,87]. The study [Harper SL, Unpublished Data] revealed no evidence of toxicity. Most recently, as part of large animal study (pig) examining the efficacy of these nanoparticles to prevent and treat hemorrhagic shock, organ histology showed no evidence of damage due to the nanoparticles including a detailed histological examination of kidney [Cabrales P, unpublished data]. Overall the emerging picture is one in which silane-derived nanoparticles are nontoxic. The exact mechanism of elimination for both topically applied nanoparticles and systemically applied nanoparticles still needs to be explored.

## Conclusion & Future perspective

The results to date indicate that the above-described NO delivery platforms have the potential to impact both topical and systemic clinical applications. Potential topical applications include uses that cover enhanced rates of wound healing and treatment of a wide variety of topical infections by most pathogens including ESKAPE organisms, fungus and parasites such as those associated with cutaneous leishmaniasis. The ability of some nanoparticle formulations to penetrate skin and release NO in a slow sustained manner opens the door for a topical treatment of erectile dysfunction. Systemic applications could include treatment of vascular inflammatory conditions arising from hemorrhage, hemoglobinopathies, hemolytic disorders and diseases caused by NO-sensitive organisms such as Chagas disease and African sleeping sickness. In all cases, the dosing and timing will prove critical since an inflammatory cascade once fully underway is fed by NO generated by macrophages. The emerging picture is one in which NO delivered at an early enough stage within the vasculature can short circuit the inflammatory cascade.

**Executive summary**

Harnessing the therapeutic potential of nitric oxide and related bioactive forms of nitric oxide such as *S*-nitrosothiols and nitrite is a challenging but important biomedical objective.
Controlled targeted nitric oxide delivery is therapeutically desirable for both systemic and topical applications.
There are several promising strategies that are currently being developed and tested.
Promising approaches include nanoparticle delivery platforms that can ultimately result in both controlled delivery and tissue-specific targeting.
Toxicity and pharmacokinetic studies are still required before clinical translation is fully possible.
